# Healthcare Provider Awareness and Perspectives on Obesity: A Survey Across Medical Specialties

**DOI:** 10.1002/osp4.70152

**Published:** 2026-06-11

**Authors:** Fatheya Alawadi, Rahila Bhatti, Khadija Hafidh, Barbara McGowan, Soniya Rai, Alice Koechlin, Amir Mohseni, Fatih Tangi, Rachel Batterham, Sara G. I. Suliman

**Affiliations:** ^1^ Dubai Hospital Dubai UAE; ^2^ Genesis Healthcare Center Dubai UAE; ^3^ Department of Endocrinology Rashid Hospital, Dubai Health Authority Dubai UAE; ^4^ Department of Diabetes and Endocrinology Guy's and St Thomas' NHS Foundation Trust London UK; ^5^ Eli Lilly and Company Dubai UAE; ^6^ Advanced Analytics and Access Capabilities Aixial Boulogne‐Billancourt France; ^7^ Eli Lilly and Company London UK; ^8^ Imperial College London Diabetes Centre (ICLDC) Abu Dhabi UAE

**Keywords:** obesity, obesity management, perceptions

## Abstract

**Background:**

Obesity is a complex, chronic disease requiring multifactorial management; however, gaps remain between clinical guidelines and real‐world practice. Given the high prevalence of obesity in the Gulf region, understanding Healthcare Providers' (HCPs) awareness and perceived barriers is essential for better, tailored regional treatment strategies.

**Objective:**

This study aimed to address the existing knowledge gaps through exploring HCP awareness, perspectives and expectations on the complexities of obesity management across various medical specialties and seeking to refine management strategies.

**Methods:**

A cross‐sectional electronic survey was conducted among HCPs attending the joint 21st International Congress of Endocrinology (ICE 2024) and 14th Emirates Diabetes and Endocrinology Congress (EDEC) held in Dubai, United Arab Emirates (UAE). Thirteen multiple‐choice questions assessed HCPs' views on the contributing factors, barriers to effective management, and expectations for future care of obesity.

**Results:**

Of approximately 3200 attendees, 565 participated (17.7% response rate). Confidence in diagnosing obesity was high (85.1%). Body Mass Index (BMI) was used as the sole diagnostic tool by 43.9% of the respondents. The rising prevalence of obesity was attributed primarily to inappropriate dietary habits and sedentary lifestyles (60.4%). Key barriers to effective obesity management reported were the cost of treatment (52.3%) and lack of patient awareness (35.5%).To enhance the quality of obesity care, HCPs emphasized the need for improved understanding of obesity pathophysiology (64.9%) and advocated for individualized treatment approaches (49.6%).

**Conclusion:**

Addressing barriers to obesity care and enhancing HCP education are important steps toward more effective and patient‐centered obesity management.

## 1 Introduction

Countries within the Gulf Cooperation Council (GCC) exhibit some of the highest obesity rates globally, with prevalence estimates ranging from 17% to 48% among women and 8%–36% among men [1]. The health consequences of obesity are considerable, with at least 4 million people dying each year due to overweight or obesity‐related conditions [2]. Most of these deaths are potentially preventable [3]. Beyond the individual health risks, obesity also poses substantial economic challenges [4].

The WOF's 2024 World Obesity Atlas report projects a substantial increase in the global economic impact of overweight and obesity, predicting it will reach $4.32 trillion annually by 2035 [5]. The report demonstrated the urgency of improving prevention, treatment, and support measures to curb this trend, showing that more than half of the global population (51%) is expected to be living with overweight or obesity within the next 12 years if current trends continue [6]. WOF emphasizes the need for comprehensive national action plans to tackle obesity and mitigate its economic and health impacts [5, 7].

In adult obesity management, healthcare providers (HCPs) focus on lifestyle modifications, pharmacotherapy, and metabolic and bariatric surgery. Traditionally, emphasis was placed on lifestyle adjustments, but with improved understanding of obesity's complex pathophysiology, clinical guidelines now advocate for pharmacotherapy or metabolic and bariatric surgery in treating obesity and its related health issues [8, 9, 10]. Despite these guidelines, their adoption is limited, with only a quarter of patients achieving weight loss of > 5% [11]. Understanding and addressing these barriers is essential to effective management of obesity. Poor insurance coverage for obesity treatment remains a major barrier to obesity management [12].

The Awareness, Care, and Treatment in Obesity; management‐International Observation (ACTION‐IO) study showed a discrepancy in obesity perceptions and attitudes between patients and HCPs, demonstrating a need for enhanced HCP education on obesity as a multifaceted disease. This education should move beyond traditional solely lifestyle‐centric interventions to more comprehensive strategies that account for environmental, psychosocial, and physiological factors influencing obesity [9, 13]. A multidisciplinary strategy, involving nutritionists, nurses, psychologists, physiotherapists, sports therapists as well as physicians is important for improving patient outcomes [14]. Given the impacts of obesity, resource conservation is essential. Key initiatives to progress management of obesity include regulatory actions, fiscal measures, educational campaigns, and promoting healthy lifestyle choices including physical activity, sleep hygiene, increased intake of wholesome foods and reduced intake of sugar sweetened beverages and ultra processed foods, as well as access to qualified obesity healthcare workers, and evidence‐based therapies. Effective management requires collaboration between patients and HCPs for improved interventions and lifestyle changes [12, 14]. This study aimed to further explore HCP awareness, perspectives and expectations on obesity management through an electronic survey, seeking to address the existing knowledge gaps and refine management strategies.

## 2 Materials and Methods

### 2.1 Study Design

This cross‐sectional, non‐interventional study employed an electronic survey targeting HCPs attending the International Congress of Endocrinology (ICE 2024) and Emirates Diabetes and Endocrinology Congress (EDEC) joint event in Dubai. An invitation to complete an electronic survey was extended to all HCPs present at the congress to evaluate their awareness and perspectives on obesity management. Participants were required to scan a QR code with their smartphones that redirected them to the survey. Before starting the survey, electronic consent was obtained from each participant. The survey responses were collected by IQVIA through IQVIA's Decipher Electronic Survey Platform, accessible only through a unique link (URL) provided to the invited participants, ensuring that each could only partake once. The survey comprised 13 multiple choice questions (survey questions could be found in supplementary material) designed to elicit HCPs' views on the contributing factors, barriers to effective management, and expectations for future care of obesity (See Suppl. Material for full survey). Conducted in English, the survey underwent thorough review and quality control checks prior to its distribution to ensure the understandability of the questions in terms of language, clarity, and consistency.

The study was approved by the Dubai Scientific Research Ethics Committee (DSREC, Ref: DSREC‐02/2024_09) and conducted in accordance with the Declaration of Helsinki, Good Pharmacoepidemiology Practices, and with electronic informed consent obtained from all participants.

### 2.2 Study Population

All registered HCPs attending the ICE 2024 and EDEC Joint Event in Dubai were invited to participate in the survey (*n* = 3200). The target population comprised a diverse group of specialists actively engaged in obesity management and its related comorbidities, including endocrinologists, family physicians, internal medicine specialists, general practitioners, nutritionists or dietitians, nurses, bariatric surgeons, gastroenterologists, nephrologists, pharmacists, psychologists, and psychiatrists. Eligibility for this study was limited to any registered HCP attending the ICE 2024 & EDEC Joint Event. No compensation was provided for participation in the survey.

### 2.3 Statistical Methods and Sample Size

The study was non‐interventional and descriptive, focusing on summarizing and describing data rather than testing hypotheses or estimating effect sizes. Therefore, statistical testing, sample size considerations, and statistical power were not applicable. A total of 565 healthcare professionals (HCPs) participated in this study using a convenience sampling approach. Descriptive analysis was conducted on the data collected through the structured questionnaire. The categorical data were summarized in frequency tables as counts and proportions of the total study population, and by subgroups where appropriate. The proportion of missing data was reported for each variable. For the perception and expectation ratings questions, only complete HCP responses with all items rated were included in the analysis.

## 3 Results

### 3.1 Demographics

#### 3.1.1 The Cohort, Detailed in Table 1, Comprised Participants From Diverse Medical Specialties

A total of 565 HCPs participated in the survey and were classified by geographic practice region (Supporting Information S1: Table S1), specialty, training and role in obesity management (Table 1). The cohort comprised participants from diverse medical specialties, with nearly half (43.4%, *n* = 245) being endocrinologists, followed by nurses (21.2%, *n* = 120), internal medicine specialists (14.9%, *n* = 84), family physicians (5.8%, *n* = 33), general practitioners (2.5%, *n* = 14), and pharmacists (1.4%, *n* = 8). The cohort had fewer nephrologists, nutritionists/dietitians, bariatric surgeons, and gastroenterologists, representing < 1% (*n* ≤ 5) each. An additional 8.3% (*n* = 47) of participants were classified as 'Other', indicating a broad range of less represented specialties.

**TABLE 1:** Sample demographics and characteristics.

*N* = 565	
Specialty	
Endocrinologist	245 (43.4%)
Nurse	120 (21.2%)
Internal medicine specialist	84 (14.9%)
Family physician	33 (5.8%)
General practitioner	14 (2.5%)
Pharmacist	8 (1.4%)
Nephrologist	5 (0.9%)
Nutritionist/dietitian	4 (0.7%)
Bariatric surgeon	3 (0.5%)
Gastroenterologist	2 (0. 4%)
Other	47 (8.3%)
Received dedicated training on obesity management	
Yes	354 (62.7%)
No	211 (37.4%)
Role in the management of obesity	
I regularly see and follow up with people who have obesity in my daily clinical practice as a part of a multi‐disciplinary team	228 (40.4%)
I am actively involved in seeing and following up with people who have obesity in my daily clinical practice	126 (22.3%)
Although I am aware that obesity is a significant healthcare issue, I have not yet had the opportunity to be involved in its management	90 (15.9%)
My focus is mainly on managing the complications of obesity related to my specialty, such as hyperlipidemia, hypertension, T2DM, etc.	76 (13.5%)
I provide consultation and support for the management of people with obesity, but I am not primarily accountable for their care	45 (8.0%)

Training on obesity management was reported by 62.7% of the HCPs (*n* = 354). (Training by specialty is presented in Supporting Information S1: Table S2). Of the HCPs surveyed, 40.4% (*n* = 228) participated in obesity management, 22.3% (*n* = 126) were actively involved in follow‐ups within their practice, 13.5% (*n* = 76) focused on managing obesity‐related complications specific to their specialties, 8.0% (*n* = 45) provided consultation and support without being the primary caregiver, and 15.9% (*n* = 90) were not engaged in the management of obesity (Table 1).

### 3.2 Interpretation

Regarding confidence in diagnosing obesity, 85.1% (*n* = 481) reported confidence in their diagnostic capabilities. The study further explored the diagnostic tools preferred by HCPs for obesity. A total of 43.9% (*n* = 248) identified Body Mass Index (BMI) as the sole diagnostic tool. An integrative approach, employing BMI in conjunction with anthropometrics, body composition, and staging criteria, was selected by 13.6% (*n* = 77) of the participants. Utilization of other tool combinations was also reported (Table 2; see Supporting Information S1: Tables S3.1 and S3.2 for an analysis of diagnostic modalities across specialties and regions, respectively).

**TABLE 2:** Perceptions.

	*N* = 565
According to your own experience, do you feel confident in diagnosing obesity?	
Yes	481 (85.1%)
No	30 (5.3%)
Not sure	54 (9.6%)
Tools needed to diagnose obesity	
BMI	248 (43.9%)
Anthropometrics	7 (1.2%)
Body composition	11 (2.0%)
Staging criteria	12 (2.1%)
BMI + anthropometrics	33 (5.8%)
BMI + body composition	53 (9.4%)
BMI + staging criteria	34 (6.0%)
Anthropometrics + body composition	3 (0.5%)
Body composition + staging criteria	1 (0.2%)
BMI + anthropometrics + body composition	43 (7.6%)
BMI + anthropometrics + staging criteria	17 (3.0%)
BMI + body composition + staging criteria	26 (4.6%)
BMI + anthropometrics + body composition + staging criteria	77 (13.6%)
Tools needed to diagnose obesity (by tool)^a^	
BMI	531 (94.0%)
Anthropometrics	180 (31.9%)
Body composition	214 (37.9%)
Staging criteria	167 (29.6%)
Perceived greatest contributor to the increased prevalence and impact of obesity	
Inappropriate food consumption and sedentary lifestyle	341 (60.4%)
Lack of recognition of obesity as a disease	88 (15.6%)
Genetic predisposition	48 (8.5%)
Lack of awareness among HCPs and society	32 (5.7%)
Lack of fundamental knowledge and training about the proper management of obesity	31 (5.5%)
Lack of appropriate treatment options	25 (4.4%)

^a^ Counted using individual diagnostic tool, multiple selection. Percentage calculated over the total number of HCPs that is using *N* = 565.

Assessing the perceived factors contributing to the rising prevalence and impact of obesity, the majority of HCPs (60.4%, *n* = 341) identified inappropriate food consumption and sedentary lifestyle as the primary causes. The lack of recognition of obesity as a disease was also considered an important factor by 15.6% (*n* = 88) of respondents, while genetic predisposition was noted by 8.5% (*n* = 48). Other contributory factors receiving acknowledgment included a lack of awareness among HCPs and society (5.7%, *n* = 32), insufficient knowledge and training on obesity management (5.5%, *n* = 31), and a deficiency of suitable treatment options (4.4%, *n* = 25). (Factors contributing to the rising prevalence across specialties and geographical regions are presented in Supporting Information S1: Tables S4.1 and S4.2).

### 3.3 Perception and Expectations Ratings Questions

**FIGURE 1:**
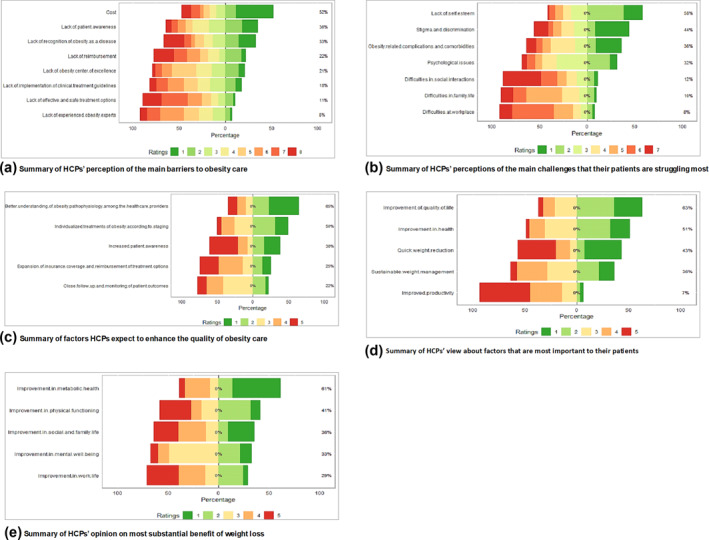
(a–e) The Likert chart displays responses to perception questions where participants arranged items from most relevant to least relevant. Green represents the highest relevance, and red represents the least. Each horizontal bar corresponds to a survey item segmented to show response proportions. The length of each colored segment within the bars reflects the number of respondents selecting that rating. The percentages on the right side represent the highest two relevance scores, indicating the proportion of respondents who rated these items as highly relevant.

“Participants prioritized barriers and challenges by ranking items from most to least relevant (Figure 1a). While the aggregate data are discussed below, a breakdown of barriers to obesity care overall and across different geographical regions is provided in Supporting Information S1: Tables S5 and S5.1, respectively. Regarding barriers to obesity care (*n* = 197), the most important barriers identified were the high cost of treatment (52.3%) and lack of patient awareness (35.5%). Conversely, lack of reimbursement (21.8%) and the absence of effective, safe treatment options (20.3%) were rated as the least relevant barriers.

When assessing the challenges faced by patients (*n* = 250), respondents reported that the psychosocial issues were most relevant. Lack of self‐esteem (58.4%) and exposure to stigma and discrimination (44.4%) were cited as the top challenges. The challenges perceived by patients are illustrated in Figure 1b, with overall, regional, and specialty data available in Supporting Information S1: Tables S6, S6.1, and S6.2, respectively.

### 3.4 Factors HCPs Expect Can Enhance the Quality of Obesity Care

To enhance the quality of obesity care (*n* = 282), HCPs emphasized the need for clinical education and personalization. A better understanding of obesity pathophysiology (64.9%) and individualized treatment based on staging (49.6%) were seen as the most effective drivers for improvement. Interestingly, expanding insurance coverage was considered the least relevant factor in this context, as shown in Figure 1c and Supporting Information S1: Table S7.

### 3.5 Priorities of Patients as Perceived by HCPs

A total of 276 HCPs rated all factors regarding patient priorities. The most relevant factors identified were the improvement of quality of life and improvement in health as rated by 63.0% and 51.1% of participants, respectively. In contrast, improved productivity was not rated highly.

In terms of patient priorities (*n* = 276), HCPs perceived that patients value improvements in quality of life (63.0%) and general health (51.1%) most highly, with improved productivity rated as less important (Figure 1d; Supporting Information S1: Table S8). Regional variations in perceived patient priorities are detailed in Supporting Information S1: Table S8.1. Finally, regarding the benefits of weight loss (*n* = 340), opinions varied; however, improvement in metabolic health (61.2%) was rated as the most substantial benefit, whereas improvement in physical functioning (31.2%) was viewed as less important (Figure 1e, Supporting Information S1: Table S9). For a breakdown of opinions by specialty, refer to Supporting Information S1: Table S9.1.

### 3.6 HCP View on What Future Holds for Obesity Care

Participants expressed general optimism regarding the future landscape of obesity care (*n* = 565). Approximately 61.8% anticipated that obesity would be more broadly accepted as a disease, and 63.9% predicted that treatments would become more accessible. Nevertheless, challenges remained; 37.0% foresaw the prevalence of obesity continuing to surge, although nearly half (47.4%) expected coordinated efforts to mitigate this trend.

## 4 Discussion

The present study revealed insights into HCPs' interpretations and perspectives on obesity management across various medical specialties. The study findings demonstrated a high confidence level among HCPs in diagnosing obesity, predominantly leveraging BMI as the principal diagnostic criterion. This reliance on BMI alone, reported by approximately 43.9% of the respondents, was consistent with prevailing clinical practice in the countries surveyed. Newer clinical guidelines advocate the inclusion of anthropometric measures such as waist circumference (WC) and waist‐to‐height ratio (WtHt) as complementary tools for a more comprehensive assessment of obesity. Implementation of these tools in routine practice may require time and additional education to ensure their widespread adoption [15].

The perceived primary drivers of obesity's rising prevalence, improper dietary habits and sedentary lifestyles, as identified by participants, resonated with the WHO assertion that these factors are major global health challenges. The WHO has indicated the rising impact of processed foods, urbanization, and lifestyle changes, which led to an increased consumption of high‐energy, high‐fat, sugary, and salty foods, contributing substantially to the global obesity burden.

Only a small proportion of respondents (8.5%) recognized genetics as a potential contributor to obesity. This low recognition persisted despite the American Medical Association designating obesity as a disease over a decade ago (2013) [16, 17]. This disconnect between medical consensus and provider perspectives highlights an important gap in messaging or awareness. This shows a gap in understanding the multifactorial nature of obesity and demonstrated the importance of enhancing education on its genetic and pathophysiological underpinnings [18].

HCPs identified a clear need for better pathophysiological understanding and individualized treatment. This suggests a shift toward more nuanced, patient‐centric care [19, 20]. This research further contributes to the discourse on healthcare system readiness to tackle obesity by demonstrating the main barriers to obesity care, such as high treatment costs and lack of patient awareness. The findings emphasize the challenges faced by patients, including stigma, discrimination, and low self‐esteem, and emphasize the need to enhance the quality of obesity care through a better understanding of obesity pathophysiology and individualized treatment approaches. This aligns with the WOF's urgent call for enhanced education and training in obesity management as part of their global guidelines [20].

While these clinical and patient‐centered approaches are essential, addressing systemic barriers, such as insurance expansion, is equally essential. Although this factor was rated lower in the survey, it remains fundamental for reducing financial obstacles to obesity treatment. Expanding reimbursement policies would support equitable access to advanced care options and help bridge the gap between clinical advancements and patient accessibility. This aligns with global calls for healthcare systems to prioritize comprehensive and sustainable strategies in managing the obesity epidemic.

Policies that support the inclusion of obesity treatments in insurance coverage could alleviate some of the economic burdens faced by patients, which is a major barrier reported in this study. While the survey revealed a relatively low response for reimbursed medication as an option to improve the quality of care, this may reflect prioritization of immediate clinical needs over systemic reforms. Addressing financial barriers through expanded reimbursement remains essential for ensuring equitable access to advanced treatments and fostering long‐term improvements in obesity management [20].

The identification of cost as a primary barrier in this study aligns with the recent real‐world evidence demonstrating the fragility of obesity care. Gasoyan et al. demonstrated that 'high cost or insurance‐related issues' are the leading cause of treatment discontinuation, accounting for nearly half of all patients who stop therapy within the first year [21]. This suggests that financial barriers do not merely prevent access but actively undermine the long‐term persistence required for effective chronic disease management [21, 22]. This reluctance to expand coverage may also stem from deep‐seated systemic biases; the University of Michigan group found that insurance coverage decisions are often hindered by a perception among payers that obesity is a 'lifestyle choice' rather than a disease [22]. To address these complexities, care models such as the 'Weight Navigation Program' which embeds specialists to help patients navigate specific insurance and cost hurdles have shown promise in improving access to evidence‐based therapies [23, 24].

The identification of 'lack of self‐esteem' (58%) as the most important challenge reported by HCPs is a notable finding. While stigma and discrimination were also noted by 44% of respondents, the prioritization of self‐esteem appears at odds with the broader body of literature on the psychosocial aspects of obesity, which typically emphasizes external drivers such as weight stigma and structural discrimination over internal psychological states [9, 20]. This discrepancy suggests that respondents may harbor knowledge gaps regarding the fundamental psychosocial complexities of obesity care. This reflects a potential delay in the adoption of evolving global scientific standards within the region, showing a disparity between current regional practice and evidence‐based medicine. These results reinforce the urgent need for greater education and training for physicians and allied health professionals [19]. Addressing this gap is essential to move beyond anecdotal perceptions and provide the comprehensive, evidence‐based care required to address both the biological and psychosocial dimensions of the disease.

Despite participants' high diagnostic confidence, this study highlighted major gaps in comprehensive obesity management, similar to the ACTION‐IO study [25]. Key barriers identified included high treatment costs, lack of patient awareness, stigma and discrimination, low self‐esteem, and psychological issues. Addressing these barriers requires fostering environments within healthcare systems that promote comprehensive and compassionate obesity care.

Despite 85% of HCPs reporting they were confident in diagnosing obesity, the reliance on BMI as the sole assessment tool by 43% reveals a gap in awareness regarding the complexity of obesity care, which shows the need for more comprehensive training.

As for what the future holds for obesity care, 61.8% of HCPs believed obesity would be more widely accepted as a disease, and 63.9% predicted improved accessibility to obesity treatments. However, 37.0% expected the prevalence of obesity to continue rising, while 47.4% anticipated coordinated efforts to reduce it. These views align with the literature advocating for broader recognition and accessibility of obesity care and reflect optimism for more effective management strategies [20, 26].

The limitations of this study included the potential lack of generalizability to the wider HCP population. With a response rate of approximately 17.7%, there is a potential for non‐response bias; participants who chose to complete the survey may have had a heightened interest in or awareness of obesity management compared to non‐participants. Difficulties in interpreting results due to inadequate respondent numbers in certain specialties and regions necessitate cautious interpretation of the findings.

It should also be noted that the rating questions did not require participants to rate all items, allowing for incomplete responses. This decision aimed to enhance participant engagement given the time constraints of the conference setting. Although this approach may have increased overall participation, it resulted in many respondents rating only the items they deemed most relevant, potentially leading to incomplete data across rating questions.

## 5 Conclusion

Although the surveyed healthcare providers (HCPs) expressed robust confidence in diagnosing obesity, the findings demonstrated a disconnect between this self‐perception and the complexities of evidence‐based care, particularly regarding the psychosocial and systemic drivers of the disease. HCPs identified inappropriate dietary habits and sedentary lifestyles as primary contributors to the rising prevalence of obesity. These behaviors should be understood within the broader context of systemic, environmental, and societal influences, such as urbanization, the availability of ultra‐processed foods, and limited opportunities for physical activity, rather than as individual shortcomings.

The selective nature of the survey respondents, primarily HCPs attending an international obesity‐focused congress, reflects a group likely to have heightened awareness of obesity management challenges. They emphasized the need for improved understanding of obesity pathophysiology and individualized treatment approaches to enhance care quality. Enhancing HCP education, utilizing multidisciplinary strategies, and implementing policy reforms are key to more effective and patient‐centered care.

Future research should explore longitudinal changes in HCP perspectives, evaluate multidisciplinary care models across diverse settings, and integrate patient viewpoints for a comprehensive understanding of obesity management. Further efforts should also aim to understand healthcare provider attitudes and identify best practices for cultivating healthcare environments that are both inclusive and compassionate. By aligning these strategies with global initiatives and evidence‐based practices, the healthcare community can make meaningful progress in addressing the obesity epidemic.

## Author Contributions

B.M., F.A., F.T., and S.R. have made contributions to the conception of the work. F.A., F.T., R.B.A., R.B.H., S.S., and S.R. have made contributions to the design of the work. FA has made contributions to the acquisition of data for the work. A.M., F.A., F.T., K.H., and S.R. have made contributions to the analysis of data for the work. A.K., A.M., B.M., F.A., F.T., K.H., R.B.H., S.S., and S.R. have made contributions to the interpretation of data for the work. AM, BM, FT, and SR have made contributions to drafting the work. A.K., A.M., F.A., F.T., K.H., R.B.A., R.B.H., S.S., and S.R. have made contributions to the revision of the manuscript for important intellectual content.

## Funding

This study was fully sponsored by Eli Lilly. Data collection was undertaken by IQVIA as part of an independent survey. Eli Lilly and Company did not influence the original survey through either contribution to the design of questionnaires or data collection. Editorial/medical writing support was provided by IQVIA and funded by Eli Lilly. This survey and the analysis were funded by Eli Lilly and Company.

## Ethics Statement

The survey was planned, conducted and reported in accordance with the Declaration of Helsinki. Dubai Scientific Research Ethics Committee (DSREC, Ref: DSREC‐02/2024_09).

## Consent

All participants provided written informed consent for the use of their anonymized and aggregated data.

## Conflicts of Interest

Alice Koechlin is an employee of Aixial and a contractor at Eli Lilly and Company. Amir Mohseni, Fatih Tangi, Rachel Batterham, and Soniya Rai are employees and minor shareholders of Eli Lilly and Company.

Fatheya Alawadi, Rahila Bhatti, and Sara Suliman have no conflicts of interest to declare.

Rachel Batterham's institutions received grants from the National Institute of Health and Care Research, Sir Jules Thorn Biomedical Trust, Rosetrees Trust, and NovoNordisk Investigator Award and received materials and drugs fom NovoNordisk (Liraglutide and placebo) for an investigator‐initiated trial. She received consulting fees from ViiV, Novo Nordisk, Gila Therapeutics, Epitome, Eli Lilly, and Pfizer. She received payment or honoraria for lectures, presentations, speakers bureaus, manuscript writing or educational events from ViiV, Novo Nordisk, Eli Lilly, International Medical Press, and Medscape. She received support for attending meetings and/or travel from Novo Nordisk and Eli Lilly and participated on a Data Safety Monitoring Board or Advisory Board for ViiV, Novo Nordisk, Gila Therapeutics, Eli Lilly, and Pfizer. She is the Chair of the Royal College of Physicians' Nutrition and Weight Management Advisory Group, Trustee and Chair of the Obesity Empowerment Network UK, Council Member of British Obesity and Metabolic Surgery Society, Trustee for Association for the Study of Obesity, Royal College of Physicians' Special Advisory for Obesity, Member of the National Bariatric Surgery Society Registry Committee, Member of the National Institute or Clinical Excellence Weight Management Committee, Member of the European Society for Endocrinology Clinical Committee, and a Member of the Royal College of Physicians' Health Inequalities Advisory Group (unpaid roles).

Khadija Hafidh’ institution has received payments from NovoNordisk, Eli Lilly, MSD, Servier, Pfizer, Roche, Novartis, Boehringer Ingelheim, and AstraZeneca.

Barbara McGowan's institution has received research grant support from NovoNordisk. She served in an advisory capacity for NovoNordisk, J&J Ethicon, Eli Lilly, Pfizer, and AstraZeneca and engaged in educational work with Eli Lilly, NovoNordisk, Boehringer Ingelheim, Janssen, Sanofi, AstraZeneca, and MSD. She is a board member and shareholder at Reset Health.

## Supporting information


Supporting Information S1

## Data Availability

The data that support the findings of this study are available on request from the corresponding author. The data are not publicly available due to privacy or ethical restrictions.
