# Supplementary material for: Healthcare Provider Awareness and Perspectives on Obesity: A Survey Across Medical Specialties
Source: Obes Sci Pract. 2026 Jun 11;12(3):e70152. doi: 10.1002/osp4.70152 (PMC13260700; doi:10.1002/osp4.70152)
Supplement: Supplementary file 1 — Supporting Information S1 [file OSP4-12-e70152-s001.pdf]

Healthcare Provider Awareness and Perspectives on Obesity: A Survey Across Medical Specialties

Authors:

Fatheya Alawadi[a], Rahila Bhatti[b], Khadija Hafidh[c], Barbara McGowan[d], Soniya Rai[e], Alice Koechlin[f], Amir Mohseni[g], Fatih Tangi[h*], Rachel Batterham[i], Sara G. I. Suliman[j]

[a] ffAlawadi@dubaihealth.ae, Dubai Hospital, Dubai, United Arab Emirates

[b] rsbhatti91@hotmail.com, Genesis Healthcare Center, Dubai, United Arab Emirates

[c] khadija.hafidh@gmail.com, Department of Endocrinology, Rashid Hospital, Dubai Health Authority, Dubai, United Arab Emirates

[d] Barbara.McGowan@gstt.nhs.uk, Department of Diabetes and Endocrinology, Guy's and St Thomas' NHS Foundation Trust, London, UK

[e] rai_soniya@lilly.com, Eli Lilly and Company, Dubai, United Arab Emirates

[f] alice.koechlin@network.lilly.com, Advanced Analytics and Access Capabilities, Aixial, France

[g] amirabbas.mohsenizonoozi@lilly.com, Eli Lilly and Company, London, UK

[h] tangi_fatih@lilly.com, Eli Lilly and Company, Dubai, United Arab Emirates

[i] rachel.batterham@lilly.com, Eli Lilly and Company, London, UK

[j] ssuliman@icldc.ae, Imperial College London Diabetes Centre (ICLDC), Abu Dhabi, United Arab Emirates

[*]**Corresponding Author**:

Full Name: Fatih Tangi

Position: Medical Director

Section: Medical Affairs

Organization/Address: Eli Lilly (Suisse) S.A. Dubai Health Care City, PO Box 25319

City: Dubai

Country: United Arab Emirates

Phone: +971585316061

Email: tangi_fatih@lilly.com

HCP Questionnaire

Definitive Questions

1. **Please choose your country/region of practice:**

   - UAE
   - East Asia and Pacific
   - Europe and Central Asia
   - Latin America and Caribbean
   - Middle East and North Africa (MENA)
   - North America, South Asia
   - Sub-Saharan Africa

2. **What is your specialty?**

   - Endocrinologist
   - Family Physician
   - Internal Medicine Specialist
   - General Practitioner
   - Nutritionist/Dietitian
   - Nurse
   - Bariatric surgeon
   - Gastroenterologist
   - Nephrologist
   - Pharmacist
   - Psychologist/Psychiatrist
   - Other

3. **I have received dedicated training on obesity management:**

   - Yes
   - No

4. **To what extent do you play an active role in the management of obesity?**

   - I regularly see and follow up with people who have obesity in my daily clinical practice as a part of a multi-disciplinary team.
   - I am actively involved in seeing and following up with people who have obesity in my daily clinical practice.
   - I provide consultation and support for the management of people with obesity, but I am not primarily accountable for their care.

- My focus is mainly on managing the complications of obesity related to my specialty, such as hyperlipidemia, hypertension, T2DM, etc.
- Although I am aware that obesity is a significant healthcare issue, I have not yet had the opportunity to be involved in its management.

Perceptions

5. **According to your own experience, do you feel confident in diagnosing obesity?**

   - Yes
   - No
   - Not sure

6. **What tools would you need to diagnose obesity? (Please choose all that are applicable)**

   - BMI
   - Anthropometrics
   - Body Composition
   - Staging Criteria

7. **What are the main barriers to obesity care? (please put in order from most relevant to less relevant)**

   - Cost
   - Lack of reimbursement
   - Lack of implementation of clinical treatment guidelines
   - Lack of patient awareness
   - Lack of obesity center of excellence
   - Lack of experienced obesity experts
   - Lack of effective and safe treatment options
   - Lack of recognition of obesity as a disease

8. **What do you think are the main challenges that your patients are struggling with the most? (please put in order from most relevant to less relevant)**

   - Stigma and discrimination
   - Lack of self-esteem
   - Psychological issues
   - Obesity-related complications & co-morbidities
   - Difficulties in family life
   - Difficulties at the workplace
   - Difficulties in social interactions

9. **What is the greatest contributor to the increased prevalence and impact of obesity?**

- Inappropriate food consumption and sedentary lifestyle
- Lack of recognition of obesity as a disease
- Lack of awareness among HCPs and society
- Lack of fundamental knowledge and training about the proper management of obesity
- Lack of appropriate treatment options
- Genetic predisposition

Expectations

10. **Which of the following could enhance the quality of obesity care? (please put in order from most relevant to less relevant)**

- Better understanding of obesity pathophysiology among healthcare providers
- Individualized treatments of obesity according to staging
- Close follow-up and monitoring of patient outcomes
- Expansion of insurance coverage and reimbursement of treatment options
- Increased patient awareness

11. **Which of the following is the most important to your patients? (please put in order from most relevant to less relevant)**

- Quick weight reduction
- Improvement of quality of life
- Improvement in health
- Sustainable weight management
- Improved productivity

12. **What does the future hold for obesity care?**

- Will be more widely accepted as a disease
- Obesity treatment will be more accessible to more individuals
- The speed of increasing prevalence will continue to surge
- There will be coordinated efforts to reduce the prevalence of obesity

13. **In your opinion as a healthcare professional, what is the most substantial benefit of weight loss? (please put in order from most relevant to less relevant)**

- Improvement in social and family life

- Improvement in work life
- Improvement in mental well-being
- Improvement in metabolic health
- Improvement in physical functioning

Table S1: Regional Distribution of participants

N=565	
**Country**	
UAE	259 (45.8%)
Middle East and North Africa (MENA)	109 (19.3%)
East Asia and Pacific	86 (15.2%)
North America, South Asia	48 (8.5%)
Europe and Central Asia	42 (7.4%)
Sub-Saharan Africa	17 (3.0%)
Latin America and Caribbean	4 (0.7%)

Table S2: Summary of HCPs basis whether they received dedicated training on obesity management by Specialties

**Specialty of HCPs**	**Received dedicated training on obesity management***		
	**Overall****	**Yes**	**No**
**N**	**354**	**211**	**565**
**Endocrinologist**	245 (43.36%)	185 (75.51%)	60 (24.49%)
**Family Physician**	33 (5.84%)	22 (66.67%)	11 (33.33%)
**Internal Medicine Specialist**	84 (14.87%)	51 (60.71%)	33 (39.29%)
**General Practitioner**	14 (2.48%)	7 (50.00%)	7 (50.00%)
**Nutritionist/Dietitian**	4 (0.71%)	3 (75.00%)	1 (25.00%)
**Nurse**	120 (21.24%)	59 (49.17%)	61 (50.83%)
**Bariatric surgeon**	3 (0.53%)	2 (66.67%)	1 (33.33%)
**Gastroenterologist**	2 (0.35%)	0 (0.00%)	2 (100.00%)
**Nephrologist**	5 (0.88%)	1 (20.00%)	4 (80.00%)
**Pharmacist**	8 (1.42%)	3 (37.50%)	5 (62.50%)
**Other**	47 (8.32%)	21 (44.68%)	26 (55.32%)

**Percentage calculated within specialty*

*** Percentage calculated within column*

Table S3.1: Summary of tools used to diagnose obesity by specialty

	Endocrinologist	Internal Medicine Specialist	Family Physician	General Practitioner & and Others	Overall
**N**	**245**	**84**	**33**	**203**	**565**
**BMI**	82 (33.47%)	39 (46.43%)	17 (51.52%)	110 (54.19%)	248 (43.89%)
**Anthropometrics**	3 (1.22%)	2 (2.38%)	0 (0.00%)	2 (0.99%)	7 (1.24%)
**Body Composition**	7 (2.86%)	1 (1.19%)	0 (0.00%)	3 (1.48%)	11 (1.95%)
**Staging Criteria**	6 (2.45%)	2 (2.38%)	3 (9.09%)	1 (0.49%)	12 (2.12%)
**BMI + Anthropometrics**	23 (9.39%)	5 (5.95%)	0 (0.00%)	5 (2.46%)	33 (5.84%)
**BMI + Body Composition**	18 (7.35%)	8 (9.52%)	2 (6.06%)	25 (12.32%)	53 (9.38%)
**BMI + Staging Criteria**	12 (4.90%)	10 (11.90%)	3 (9.09%)	9 (4.43%)	34 (6.02%)
**Anthropometrics + Body Composition**	2 (0.82%)	0 (0.00%)	0 (0.00%)	1 (0.49%)	3 (0.53%)
**Body Composition + Staging Criteria**	1 (0.41%)	0 (0.00%)	0 (0.00%)	0 (0.00%)	1 (0.18%)
**BMI + Anthropometrics + Body Composition**	28 (11.43%)	2 (2.38%)	1 (3.03%)	12 (5.91%)	43 (7.61%)
**BMI + Anthropometrics + Staging Criteria**	14 (5.71%)	2 (2.38%)	0 (0.00%)	1 (0.49%)	17 (3.01%)
**BMI + Body Composition + Staging Criteria**	9 (3.67%)	4 (4.76%)	4 (12.12%)	9 (4.43%)	26 (4.60%)
**BMI + Anthropometrics + Body Composition + Staging Criteria**	40 (16.33%)	9 (10.71%)	3 (9.09%)	25 (12.32%)	77 (13.63%)
**Tools used in diagnosis by tool***					
**BMI**	226 (92.24%)	30 (90.91%)	79 (94.05%)	196 (96.55%)	531 (93.98%)
**Anthropometrics**	110 (44.9%)	4 (12.12%)	20 (23.81%)	46 (22.66%)	180 (31.86%)
**Body Composition**	105 (42.86%)	10 (30.3%)	24 (28.57%)	75 (36.95%)	214 (37.88%)
**Staging Criteria**	82 (33.47%)	13 (39.39%)	27 (32.14%)	45 (22.17%)	167 (29.56%)

Based on all possible of combinations of choices selected are enlisted and summarized

General Practitioner & Others includes all other specialties and the ones mentioned as "other"

**Counted using individual diagnostic tool, multiple selection. Percentage calculated over the total number of HCPs i.e. using N=565*

Table S3.2: Summary of tools used to diagnose obesity by HCPS by geographical region

	Regions							
**Variable**	**UAE**	**East Asia and Pacific**	**Europe and Central Asia**	**Latin America and Caribbean**	**Middle East and North Africa (MENA)**	**North America, South Asia**	**Sub-Saharan Africa**	**Overall**
**N**	**259**	**86**	**42**	**4**	**109**	**48**	**17**	**565**
**BMI**	132 (50.97%)	36 (41.86%)	9 (21.43%)	1 (25.00%)	43 (39.45%)	21 (43.75%)	6 (35.29%)	248 (43.89%)
**Anthropometrics**	3 (1.16%)	0 (0.00%)	1 (2.38%)	0 (0.00%)	2 (1.83%)	1 (2.08%)	0 (0.00%)	7 (1.24%)
**Body Composition**	5 (1.93%)	1 (1.16%)	1 (2.38%)	0 (0.00%)	2 (1.83%)	1 (2.08%)	1 (5.88%)	11 (1.95%)
**Staging Criteria**	4 (1.54%)	0 (0.00%)	0 (0.00%)	0 (0.00%)	5 (4.59%)	1 (2.08%)	2 (11.76%)	12 (2.12%)
**BMI + Anthropometrics**	7 (2.70%)	5 (5.81%)	5 (11.90%)	1 (25.00%)	6 (5.50%)	6 (12.50%)	3 (17.65%)	33 (5.84%)
**BMI + Body Composition**	30 (11.58%)	10 (11.63%)	5 (11.90%)	2 (50.00%)	5 (4.59%)	1 (2.08%)	0 (0.00%)	53 (9.38%)
**BMI + Staging Criteria**	16 (6.18%)	4 (4.65%)	2 (4.76%)	0 (0.00%)	10 (9.17%)	1 (2.08%)	1 (5.88%)	34 (6.02%)
**Anthropometrics + Body Composition**	1 (0.39%)	1 (1.16%)	1 (2.38%)	0 (0.00%)	0 (0.00%)	0 (0.00%)	0 (0.00%)	3 (0.53%)
**Body Composition +**	0 (0.00%)	0 (0.00%)	0 (0.00%)	0 (0.00%)	1 (0.92%)	0 (0.00%)	0 (0.00%)	1 (0.18%)

**Staging Criteria**								
**BMI + Anthropometrics + Body Composition**	11 (4.25%)	12 (13.95%)	8 (19.05%)	0 (0.00%)	7 (6.42%)	3 (6.25%)	2 (11.76%)	43 (7.61%)
**BMI + Anthropometrics + Staging Criteria**	5 (1.93%)	4 (4.65%)	1 (2.38%)	0 (0.00%)	3 (2.75%)	4 (8.33%)	0 (0.00%)	17 (3.01%)
**BMI + Body Composition + Staging Criteria**	19 (7.34%)	1 (1.16%)	0 (0.00%)	0 (0.00%)	6 (5.50%)	0 (0.00%)	0 (0.00%)	26 (4.60%)
**BMI + Anthropometrics + Body Composition + Staging Criteria**	26 (10.04%)	12 (13.95%)	9 (21.43%)	0 (0.00%)	19 (17.43%)	9 (18.75%)	2 (11.76%)	77 (13.63%)
**Tools used in diagnosis by tool***								
**BMI**	246 (94.98%)	84 (97.67%)	39 (92.86%)	4 (100%)	99 (90.83%)	45 (93.75%)	14 (82.35%)	531 (93.98%)
**Anthropometrics**	53 (20.46%)	34 (39.53%)	25 (59.52%)	1 (25%)	37 (33.94%)	23 (47.92%)	7 (41.18%)	180 (31.86%)
**Body Composition**	92 (35.52%)	37 (43.02%)	24 (57.14%)	2 (50%)	40 (36.7%)	14 (29.17%)	5 (29.41%)	214 (37.88%)
**Staging Criteria**	70 (27.03%)	21 (24.42%)	12 (28.57%)	0 (0%)	44 (40.37%)	15 (31.25%)	5 (29.41%)	167 (29.56%)

Based on all possible combinations of choices selected are enlisted and summarized

**Counted using individual diagnostic tool, multiple selection. Percentage calculated over the total number of HCPs i.e. using N=565*

Table S4.1: HCPs' views regarding the greatest contributor to the increased prevalence and impact of obesity by Specialty

Variable	Endocrinologist	Family Physician	Internal Medicine Specialist	General Practitioner & Others	Overall
**N**	**245**	**33**	**84**	**203**	**565**
**Inappropriate food consumption and sedentary lifestyle**	159 (64.90%)	17 (51.52%)	51 (60.71%)	114 (56.16%)	341 (60.35%)
**Lack of recognition of obesity as a disease**	39 (15.92%)	5 (15.15%)	12 (14.29%)	32 (15.76%)	88 (15.58%)
**Lack of awareness among HCPs and society**	8 (3.27%)	4 (12.12%)	3 (3.57%)	17 (8.37%)	32 (5.66%)
**Lack of fundamental knowledge and training about the proper management of obesity**	11 (4.49%)	0 (0.00%)	6 (7.14%)	14 (6.90%)	31 (5.49%)
**Lack of appropriate treatment options**	10 (4.08%)	2 (6.06%)	0 (0.00%)	13 (6.40%)	25 (4.42%)
**Genetic predisposition**	18 (7.35%)	5 (15.15%)	12 (14.29%)	13 (6.40%)	48 (8.50%)

Table S4.2: HCPs' views about the greatest contributor to the increased prevalence and impact of obesity by regions.

Perceived greatest contributor to the increased prevalence and impact of obesity	Regions							
	UAE	East Asia and Pacific	Europe and Central Asia	Latin America and Caribbean	Middle East and North Africa (MENA)	North America, South Asia	Sub-Saharan Africa	Overall
N	259	86	42	4	109	48	17	565
Inappropriate food consumption and sedentary lifestyle	135 (52.12%)	60 (69.77%)	32 (76.19%)	2 (50.00%)	68 (62.39%)	35 (72.92%)	9 (52.94%)	341 (60.35%)
Lack of recognition of obesity as a disease	47 (18.15%)	7 (8.14%)	5 (11.90%)	0 (0.00%)	20 (18.35%)	7 (14.58%)	2 (11.76%)	88 (15.58%)
Lack of awareness among HCPs and society	22 (8.49%)	2 (2.33%)	0 (0.00%)	1 (25.00%)	3 (2.75%)	3 (6.25%)	1 (5.88%)	32 (5.66%)
Lack of fundamental knowledge and training about the proper management of obesity	16 (6.18%)	5 (5.81%)	1 (2.38%)	0 (0.00%)	5 (4.59%)	2 (4.17%)	2 (11.76%)	31 (5.49%)
Lack of appropriate treatment options	13 (5.02%)	7 (8.14%)	2 (4.76%)	0 (0.00%)	1 (0.92%)	0 (0.00%)	2 (11.76%)	25 (4.42%)
Genetic predisposition	26 (10.04%)	5 (5.81%)	2 (4.76%)	1 (25.00%)	12 (11.01%)	1 (2.08%)	1 (5.88%)	48 (8.50%)

percentages are by column

Table S5: Summary of HCPs' perception of the main barriers to obesity care

Variable	Overall (n=197)							
	Most Relevant to least rating							
	1	2	3	4	5	6	7	8
Cost	80 (40.61%)	23 (11.68%)	19 (9.64%)	14 (7.11%)	14 (7.11%)	7 (3.55%)	19 (9.64%)	21 (10.66%)
Lack of patient awareness	34 (17.26%)	36 (18.27%)	35 (17.77%)	35 (17.77%)	15 (7.61%)	17 (8.63%)	13 (6.6%)	12 (6.09%)
Lack of recognition of obesity as a disease	37 (18.78%)	28 (14.21%)	27 (13.71%)	19 (9.64%)	19 (9.64%)	8 (4.06%)	14 (7.11%)	45 (22.84%)
Lack of reimbursement	10 (5.08%)	34 (17.26%)	13 (6.6%)	24 (12.18%)	17 (8.63%)	28 (14.21%)	28 (14.21%)	43 (21.83%)
Lack of obesity center of excellence	13 (6.6%)	28 (14.21%)	29 (14.72%)	33 (16.75%)	52 (26.4%)	21 (10.66%)	15 (7.61%)	6 (3.05%)
Lack of implementation of clinical treatment guidelines	13 (6.6%)	22 (11.17%)	32 (16.24%)	27 (13.71%)	29 (14.72%)	35 (17.77%)	24 (12.18%)	15 (7.61%)
Lack of effective and safe treatment options	5 (2.54%)	16 (8.12%)	15 (7.61%)	16 (8.12%)	19 (9.64%)	30 (15.23%)	56 (28.43%)	40 (20.3%)
Lack of experienced obesity experts	5 (2.54%)	10 (5.08%)	27 (13.71%)	29 (14.72%)	32 (16.24%)	51 (25.89%)	28 (14.21%)	15 (7.61%)

- *A total of 197 HCPs rated all options regarding their perceptions about barriers to obesity care.*

- *The tables use a color code to indicate relevance: green for the highest relevance, transitioning through yellow for moderate relevance or with mixed responses, to red for the least relevance.*

- Percentages are by row.

Table S5.1: Summary of HCPs' perception of the main barriers to obesity care by geographical region

UAE (n=77)								
Variable	Most Relevant to least rating							
	1	2	3	4	5	6	7	8
Cost	21 (27.27%)	11 (14.29%)	9 (11.69%)	5 (6.49%)	4 (5.19%)	3 (3.9%)	11 (14.29%)	13 (16.88%)
Lack of patient awareness	15 (19.48%)	20 (25.97%)	10 (12.99%)	15 (19.48%)	3 (3.9%)	7 (9.09%)	4 (5.19%)	3 (3.9%)
Lack of recognition of obesity as a disease	16 (20.78%)	12 (15.58%)	10 (12.99%)	10 (12.99%)	6 (7.79%)	3 (3.9%)	2 (2.6%)	18 (23.38%)
Lack of reimbursement	6 (7.79%)	9 (11.69%)	6 (7.79%)	11 (14.29%)	8 (10.39%)	9 (11.69%)	12 (15.58%)	16 (20.78%)
Lack of obesity center of excellence	6 (7.79%)	9 (11.69%)	13 (16.88%)	9 (11.69%)	19 (24.68%)	10 (12.99%)	8 (10.39%)	3 (3.9%)
Lack of implementation of clinical treatment guidelines	6 (7.79%)	5 (6.49%)	16 (20.78%)	9 (11.69%)	16 (20.78%)	15 (19.48%)	6 (7.79%)	4 (5.19%)
Lack of effective and safe treatment options	3 (3.9%)	6 (7.79%)	6 (7.79%)	6 (7.79%)	8 (10.39%)	12 (15.58%)	25 (32.47%)	11 (14.29%)
Lack of experienced obesity experts	4 (5.19%)	5 (6.49%)	7 (9.09%)	12 (15.58%)	13 (16.88%)	18 (23.38%)	9 (11.69%)	9 (11.69%)

MENA (n=36)								
Variable	Most Relevant to least rating							
	1	2	3	4	5	6	7	8
Cost	23 (63.89%)	1 (2.78%)	0 (0%)	4 (11.11%)	2 (5.56%)	1 (2.78%)	2 (5.56%)	3 (8.33%)
Lack of patient awareness	5 (13.89%)	4 (11.11%)	8 (22.22%)	6 (16.67%)	5 (13.89%)	2 (5.56%)	2 (5.56%)	4 (11.11%)
Lack of recognition of obesity as a disease	5 (13.89%)	4 (11.11%)	8 (22.22%)	2 (5.56%)	6 (16.67%)	1 (2.78%)	5 (13.89%)	5 (13.89%)
Lack of reimbursement	1 (2.78%)	10 (27.78%)	1 (2.78%)	4 (11.11%)	4 (11.11%)	6 (16.67%)	5 (13.89%)	5 (13.89%)
Lack of obesity center of excellence	0 (0%)	7 (19.44%)	6 (16.67%)	6 (16.67%)	10 (27.78%)	4 (11.11%)	2 (5.56%)	1 (2.78%)
Lack of implementation of clinical treatment guidelines	1 (2.78%)	4 (11.11%)	5 (13.89%)	5 (13.89%)	2 (5.56%)	7 (19.44%)	8 (22.22%)	4 (11.11%)
Lack of effective and safe treatment options	1 (2.78%)	5 (13.89%)	1 (2.78%)	3 (8.33%)	5 (13.89%)	4 (11.11%)	5 (13.89%)	12 (33.33%)
Lack of experienced obesity experts	0 (0%)	1 (2.78%)	7 (19.44%)	6 (16.67%)	2 (5.56%)	11 (30.56%)	7 (19.44%)	2 (5.56%)

- *Only UAE and MENA regions were considered, as they had the highest number of respondents.*
- *The tables use a color code to indicate relevance: green for the highest relevance, transitioning through yellow for moderate relevance or with mixed responses, to red for the least relevance.*
- *Percentages are by row*

Table S6: Summary of HCPs' perception of the main challenges that their patients are struggling with .

Variable	Most Relevant to least rating						
	1	2	3	4	5	6	7
Lack of self esteem	50 (20%)	96 (38.4%)	42 (16.8%)	21 (8.4%)	20 (8%)	16 (6.4%)	5 (2%)
Stigma and discrimination	90 (36%)	21 (8.4%)	34 (13.6%)	34 (13.6%)	21 (8.4%)	12 (4.8%)	38 (15.2%)
Obesity related complications & co-morbidities	68 (27.2%)	23 (9.2%)	32 (12.8%)	63 (25.2%)	22 (8.8%)	16 (6.4%)	26 (10.4%)
Psychological issues	19 (7.6%)	60 (24%)	80 (32%)	38 (15.2%)	18 (7.2%)	21 (8.4%)	14 (5.6%)
Difficulties in social interactions	11 (4.4%)	18 (7.2%)	26 (10.4%)	28 (11.2%)	24 (9.6%)	42 (16.8%)	101 (40.4%)
Difficulties in family life	6 (2.4%)	18 (7.2%)	20 (8%)	45 (18%)	94 (37.6%)	35 (14%)	32 (12.8%)
Difficulties at workplace	6 (2.4%)	14 (5.6%)	16 (6.4%)	21 (8.4%)	51 (20.4%)	108 (43.2%)	34 (13.6%)

- *A total of 250 HCPs rated challenges that their patients are struggling with from most relevant (rating 1) to the least relevant (rating 7)*
- *The tables use a color code to indicate relevance: green for the highest relevance, transitioning through yellow for moderate relevance or with mixed responses, to red for the least relevance.*
- *Percentages are by row*

Table S6.1: Summary of HCPs' perception of the main challenges that their patients are struggling with by region

UAE (n=104)							
Variable	Most Relevant to least rating						
	1	2	3	4	5	6	7
Lack of self esteem	24 (23.08%)	48 (46.15%)	9 (8.65%)	6 (5.77%)	8 (7.69%)	6 (5.77%)	3 (2.88%)
Stigma and discrimination	38 (36.54%)	7 (6.73%)	15 (14.42%)	13 (12.5%)	7 (6.73%)	5 (4.81%)	19 (18.27%)
Obesity related complications & co-morbidities	24 (23.08%)	10 (9.62%)	13 (12.5%)	30 (28.85%)	9 (8.65%)	6 (5.77%)	12 (11.54%)
Psychological issues	6 (5.77%)	20 (19.23%)	38 (36.54%)	17 (16.35%)	9 (8.65%)	10 (9.62%)	4 (3.85%)
Difficulties in social interactions	5 (4.81%)	6 (5.77%)	12 (11.54%)	14 (13.46%)	11 (10.58%)	14 (13.46%)	42 (40.38%)
Difficulties in family life	4 (3.85%)	6 (5.77%)	9 (8.65%)	18 (17.31%)	40 (38.46%)	15 (14.42%)	12 (11.54%)
Difficulties at workplace	3 (2.88%)	7 (6.73%)	8 (7.69%)	6 (5.77%)	20 (19.23%)	48 (46.15%)	12 (11.54%)

MENA (n=48)							
Variable	Most Relevant to least rating						
	1	2	3	4	5	6	7
Lack of self esteem	8 (16.67%)	19 (39.58%)	9 (18.75%)	5 (10.42%)	3 (6.25%)	4 (8.33%)	0 (0%)
Stigma and discrimination	21 (43.75%)	3 (6.25%)	5 (10.42%)	7 (14.58%)	6 (12.5%)	2 (4.17%)	4 (8.33%)
Obesity related complications & co-morbidities	11 (22.92%)	3 (6.25%)	7 (14.58%)	17 (35.42%)	4 (8.33%)	2 (4.17%)	4 (8.33%)
Psychological issues	4 (8.33%)	13 (27.08%)	15 (31.25%)	5 (10.42%)	5 (10.42%)	3 (6.25%)	3 (6.25%)
Difficulties in social interactions	2 (4.17%)	4 (8.33%)	8 (16.67%)	2 (4.17%)	1 (2.08%)	8 (16.67%)	23 (47.92%)
Difficulties in family life	1 (2.08%)	4 (8.33%)	2 (4.17%)	9 (18.75%)	20 (41.67%)	7 (14.58%)	5 (10.42%)
Difficulties at workplace	1 (2.08%)	2 (4.17%)	2 (4.17%)	3 (6.25%)	9 (18.75%)	22 (45.83%)	9 (18.75%)

- *Only UAE and MENA regions were considered, as they had the highest number of respondents.*
- *The tables use a color code to indicate relevance: green for the highest relevance, transitioning through yellow for moderate relevance or with mixed responses, to red for the least relevance.*
- *Percentages are by row*

Table S6.2: Summary of HCPs' perception of the main challenges that their patients are struggling with by specialty

Endocrinologist (n=109)							
Variable	Most Relevant to least rating						
	1	2	3	4	5	6	7
Lack of self esteem	14 (12.84%)	36 (33.03%)	22 (20.18%)	15 (13.76%)	12 (11.01%)	9 (8.26%)	1 (0.92%)
Stigma and discrimination	35 (32.11%)	9 (8.26%)	16 (14.68%)	20 (18.35%)	12 (11.01%)	3 (2.75%)	14 (12.84%)
Obesity related complications & co-morbidities	39 (35.78%)	10 (9.17%)	16 (14.68%)	19 (17.43%)	5 (4.59%)	6 (5.5%)	14 (12.84%)
Psychological issues	13 (11.93%)	28 (25.69%)	31 (28.44%)	15 (13.76%)	7 (6.42%)	9 (8.26%)	6 (5.5%)
Difficulties in social interactions	4 (3.67%)	12 (11.01%)	11 (10.09%)	12 (11.01%)	12 (11.01%)	19 (17.43%)	39 (35.78%)
Difficulties in family life	1 (0.92%)	8 (7.34%)	9 (8.26%)	18 (16.51%)	39 (35.78%)	18 (16.51%)	16 (14.68%)
Difficulties at workplace	3 (2.75%)	6 (5.5%)	4 (3.67%)	10 (9.17%)	22 (20.18%)	45 (41.28%)	19 (17.43%)

Family Physician (n=18)							
Variable	Most Relevant to least rating						
	1	2	3	4	5	6	7
Lack of self esteem	4 (22.22%)	7 (38.89%)	3 (16.67%)	1 (5.56%)	1 (5.56%)	1 (5.56%)	1 (5.56%)
Stigma and discrimination	7 (38.89%)	0 (0%)	0 (0%)	4 (22.22%)	1 (5.56%)	2 (11.11%)	4 (22.22%)
Obesity related complications & co-morbidities	3 (16.67%)	4 (22.22%)	2 (11.11%)	5 (27.78%)	2 (11.11%)	1 (5.56%)	1 (5.56%)
Psychological issues	2 (11.11%)	2 (11.11%)	7 (38.89%)	3 (16.67%)	1 (5.56%)	2 (11.11%)	1 (5.56%)
Difficulties in social interactions	1 (5.56%)	1 (5.56%)	3 (16.67%)	1 (5.56%)	0 (0%)	4 (22.22%)	8 (44.44%)
Difficulties in family life	1 (5.56%)	2 (11.11%)	1 (5.56%)	2 (11.11%)	7 (38.89%)	4 (22.22%)	1 (5.56%)
Difficulties at workplace	0 (0%)	2 (11.11%)	2 (11.11%)	2 (11.11%)	6 (33.33%)	4 (22.22%)	2 (11.11%)

Internal Medicine Specialist (n=35)							
Variable	Most Relevant to least rating						
	1	2	3	4	5	6	7
Lack of self esteem	7 (20%)	15 (42.86%)	7 (20%)	2 (5.71%)	2 (5.71%)	2 (5.71%)	0 (0%)
Stigma and discrimination	14 (40%)	4 (11.43%)	6 (17.14%)	2 (5.71%)	0 (0%)	3 (8.57%)	6 (17.14%)
Obesity related complications & co-morbidities	10 (28.57%)	3 (8.57%)	2 (5.71%)	10 (28.57%)	5 (14.29%)	1 (2.86%)	4 (11.43%)
Psychological issues	1 (2.86%)	7 (20%)	11 (31.43%)	4 (11.43%)	3 (8.57%)	5 (14.29%)	4 (11.43%)
Difficulties in social interactions	2 (5.71%)	1 (2.86%)	2 (5.71%)	4 (11.43%)	4 (11.43%)	9 (25.71%)	13 (37.14%)
Difficulties in family life	1 (2.86%)	4 (11.43%)	3 (8.57%)	9 (25.71%)	11 (31.43%)	4 (11.43%)	3 (8.57%)
Difficulties at workplace	0 (0%)	1 (2.86%)	4 (11.43%)	4 (11.43%)	10 (28.57%)	11 (31.43%)	5 (14.29%)

GP and others (n=88)							
Variable	Most Relevant to least rating						
	1	2	3	4	5	6	7
Lack of self esteem	25 (28.41%)	38 (43.18%)	10 (11.36%)	3 (3.41%)	5 (5.68%)	4 (4.55%)	3 (3.41%)
Stigma and discrimination	34 (38.64%)	8 (9.09%)	12 (13.64%)	8 (9.09%)	8 (9.09%)	4 (4.55%)	14 (15.91%)
Obesity related complications & co-morbidities	16 (18.18%)	6 (6.82%)	12 (13.64%)	29 (32.95%)	10 (11.36%)	8 (9.09%)	7 (7.95%)
Psychological issues	3 (3.41%)	23 (26.14%)	31 (35.23%)	16 (18.18%)	7 (7.95%)	5 (5.68%)	3 (3.41%)
Difficulties in social interactions	4 (4.55%)	4 (4.55%)	10 (11.36%)	11 (12.5%)	8 (9.09%)	10 (11.36%)	41 (46.59%)
Difficulties in family life	3 (3.41%)	4 (4.55%)	7 (7.95%)	16 (18.18%)	37 (42.05%)	9 (10.23%)	12 (13.64%)
Difficulties at workplace	3 (3.41%)	5 (5.68%)	6 (6.82%)	5 (5.68%)	13 (14.77%)	48 (54.55%)	8 (9.09%)

- *The tables use a color code to indicate relevance: green for the highest relevance, transitioning through yellow for moderate relevance or with mixed responses, to red for the least relevance.*
- *Percentages are by row*

Table S7: Summary of HCPs' perception of factors expected to enhance the quality of obesity care.

Variable	Most Relevant to least rating				
	1	2	3	4	5
Better understanding of obesity pathophysiology among the healthcare providers	120 (42.55%)	63 (22.34%)	28 (9.93%)	34 (12.06%)	37 (13.12%)
Individualized treatments of obesity according to staging	52 (18.44%)	88 (31.21%)	73 (25.89%)	51 (18.09%)	18 (6.38%)
Increased patient awareness	64 (22.7%)	44 (15.6%)	22 (7.8%)	37 (13.12%)	115 (40.78%)
Expansion of insurance coverage and reimbursement of treatment options	34 (12.06%)	37 (13.12%)	41 (14.54%)	95 (33.69%)	75 (26.6%)
Close follow-up and monitoring of patient outcomes	12 (4.26%)	50 (17.73%)	118 (41.84%)	65 (23.05%)	37 (13.12%)

n=282 HCPs who responded to rate all options for their expectations about quality of obesity care.

- *The tables use a color code to indicate relevance: green for the highest relevance, transitioning through yellow for moderate relevance or with mixed responses, to red for the least relevance.*
- *Percentages are by row*

Table S8: Summary of HCPs' perception of factors most important to their patients

Overall (n=276)					
Variable	Most Relevant to least rating				
	1	2	3	4	5
Improvement of quality of life	75 (27.17%)	99 (35.87%)	58 (21.01%)	31 (11.23%)	13 (4.71%)
Improvement in health	52 (18.84%)	89 (32.25%)	84 (30.43%)	41 (14.86%)	10 (3.62%)
Quick weight reduction	99 (35.87%)	20 (7.25%)	18 (6.52%)	37 (13.41%)	102 (36.96%)
Sustainable weight management	41 (14.86%)	59 (21.38%)	78 (28.26%)	81 (29.35%)	17 (6.16%)
Improved productivity	9 (3.26%)	9 (3.26%)	38 (13.77%)	86 (31.16%)	134 (48.55%)

- *n=276 HCPs who responded to rate all options for their perceptions about factors that are most important to their patients.*
- *The tables use a color code to indicate relevance: green for the highest relevance, transitioning through yellow for moderate relevance or with mixed responses, to red for the least relevance.*
- *Percentages are by row*

Table S8.1: Summary of HCPs' perception of factors most important to their patients by geographical region

UAE (n=113)					
Variable	Most Relevant to least rating				
	1	2	3	4	5
Improvement of quality of life	32 (28.32%)	43 (38.05%)	21 (18.58%)	10 (8.85%)	7 (6.19%)
Improvement in health	25 (22.12%)	41 (36.28%)	34 (30.09%)	11 (9.73%)	2 (1.77%)
Quick weight reduction	36 (31.86%)	7 (6.19%)	8 (7.08%)	14 (12.39%)	48 (42.48%)
Sustainable weight management	17 (15.04%)	16 (14.16%)	33 (29.2%)	38 (33.63%)	9 (7.96%)
Improved productivity	3 (2.65%)	6 (5.31%)	17 (15.04%)	40 (35.4%)	47 (41.59%)

MENA (n=57)					
Variable	Most Relevant to least rating				
	1	2	3	4	5
Improvement of quality of life	15 (26.32%)	18 (31.58%)	12 (21.05%)	9 (15.79%)	3 (5.26%)
Improvement in health	9 (15.79%)	14 (24.56%)	15 (26.32%)	15 (26.32%)	4 (7.02%)
Quick weight reduction	26 (45.61%)	7 (12.28%)	1 (1.75%)	8 (14.04%)	15 (26.32%)
Sustainable weight management	5 (8.77%)	17 (29.82%)	20 (35.09%)	11 (19.3%)	4 (7.02%)
Improved productivity	2 (3.51%)	1 (1.75%)	9 (15.79%)	14 (24.56%)	31 (54.39%)

- *Only UAE and MENA regions were considered, as they had the highest number of respondents.*
- *The tables use a color code to indicate relevance: green for the highest relevance, transitioning through yellow for moderate relevance or with mixed responses, to red for the least relevance.*
- *Percentages are by row*

Table S9: HCPs' opinion on most substantial benefit of weight loss.

Overall (n=340)					
Variable	Most Relevant to least rating				
	1	2	3	4	5
Improvement in metabolic health	161 (47.35%)	47 (13.82%)	29 (8.53%)	84 (24.71%)	19 (5.59%)
Improvement in physical functioning	33 (9.71%)	108 (31.76%)	57 (16.76%)	36 (10.59%)	106 (31.18%)
Improvement in social and family life	90 (26.47%)	31 (9.12%)	42 (12.35%)	94 (27.65%)	83 (24.41%)
Improvement in mental well being	39 (11.47%)	72 (21.18%)	167 (49.12%)	36 (10.59%)	26 (7.65%)
Improvement in work life	17 (5%)	82 (24.12%)	45 (13.24%)	90 (26.47%)	106 (31.18%)

- *n=340 HCPs who responded to rate all options for their opinion on most substantial benefit of weight loss*
- *The tables use a color code to indicate relevance: green for the highest relevance, transitioning through yellow for moderate relevance or with mixed responses, to red for the least relevance.*
- *Percentages are by row*

Table S9.1: HCPs' opinion on most substantial benefit of weight loss.

Endocrinologist (n=149)					
Variable	Most Relevant to least rating				
	1	2	3	4	5
Improvement in metabolic health	93 (62.42%)	23 (15.44%)	5 (3.36%)	25 (16.78%)	3 (2.01%)
Improvement in physical functioning	12 (8.05%)	58 (38.93%)	30 (20.13%)	8 (5.37%)	41 (27.52%)
Improvement in social and family life	28 (18.79%)	12 (8.05%)	20 (13.42%)	49 (32.89%)	40 (26.85%)
Improvement in mental well being	14 (9.4%)	29 (19.46%)	79 (53.02%)	14 (9.4%)	13 (8.72%)
Improvement in work life	2 (1.34%)	27 (18.12%)	15 (10.07%)	53 (35.57%)	52 (34.9%)

Family Physician (n=20)					
Variable	Most Relevant to least rating				
	1	2	3	4	5
Improvement in metabolic health	8 (40%)	3 (15%)	4 (20%)	5 (25%)	0 (0%)
Improvement in physical functioning	3 (15%)	5 (25%)	3 (15%)	2 (10%)	7 (35%)
Improvement in social and family life	7 (35%)	4 (20%)	3 (15%)	2 (10%)	4 (20%)
Improvement in mental well being	1 (5%)	5 (25%)	9 (45%)	3 (15%)	2 (10%)
Improvement in work life	1 (5%)	3 (15%)	1 (5%)	8 (40%)	7 (35%)

Internal Medicine Specialist (n=56)					
Variable	Most Relevant to least rating				
	1	2	3	4	5
Improvement in metabolic health	28 (50%)	5 (8.93%)	8 (14.29%)	14 (25%)	1 (1.79%)
Improvement in physical functioning	4 (7.14%)	20 (35.71%)	9 (16.07%)	9 (16.07%)	14 (25%)
Improvement in social and family life	16 (28.57%)	3 (5.36%)	4 (7.14%)	17 (30.36%)	16 (28.57%)
Improvement in mental well being	6 (10.71%)	13 (23.21%)	26 (46.43%)	5 (8.93%)	6 (10.71%)
Improvement in work life	2 (3.57%)	15 (26.79%)	9 (16.07%)	11 (19.64%)	19 (33.93%)

GP and others (n=115)					
Variable	Most Relevant to least rating				
	1	2	3	4	5
Improvement in metabolic health	32 (27.83%)	16 (13.91%)	12 (10.43%)	40 (34.78%)	15 (13.04%)
Improvement in physical functioning	14 (12.17%)	25 (21.74%)	15 (13.04%)	17 (14.78%)	44 (38.26%)
Improvement in social and family life	39 (33.91%)	12 (10.43%)	15 (13.04%)	26 (22.61%)	23 (20%)
Improvement in mental well being	18 (15.65%)	25 (21.74%)	53 (46.09%)	14 (12.17%)	5 (4.35%)
Improvement in work life	12 (10.43%)	37 (32.17%)	20 (17.39%)	18 (15.65%)	28 (24.35%)

The tables use a color code to indicate relevance: green for the highest relevance, transitioning through yellow for moderate relevance or with mixed responses, to red for the least relevance.
